# Anatomic feasibility of using Shark Screw tendon for humeral ligament re-construction in medial and posterolateral elbow instability

**DOI:** 10.1016/j.jseint.2026.101786

**Published:** 2026-08-28

**Authors:** Lisa Hainzer, Sepp Braun, Markus Reichkendler, Helmut Weitlaner, Simon Lechner, Samuel Dollnig, Lukas F. Reissig, Stephan Pauly, Kathi Thiele

**Affiliations:** a GELENKpunkt, Specialist Practice for Orthopedics, Trauma Surgery and Sports, Traumatology, Innsbruck, Austria; b District Hospital Schwaz, Orthopedics and Traumatology, Schwaz, Austria; c Division of Anatomy, Center for Anatomy and Cell Biology, Medical University of Vienna, Vienna, Austria; d Clinic for Shoulder and Elbow Surgery, Auguste-Viktoria Clinic, Berlin, Germany; e Center for Musculoskeletal Surgery, Charité - Universitätsmedizin Berlin, Berlin, Germany

**Keywords:** Elbow, Instability, Humeral fixation, Shark Screw tendon, Basic science study, Biomechanics

## Abstract

**Background:**

Medial and posterolateral rotational instability of the elbow, often resulting from collateral ligament insufficiency, can lead to pain and functional impairment. Surgical re-construction is indicated when conservative treatment fails, yet complications such as graft failure and donor-site morbidity persist. This study investigates the anatomic and biomechanical feasibility of the Shark Screw tendon as a representative human allogeneic bone screw for humeral ligament fixation in cases of elbow instability.

**Methods:**

Eight fresh-frozen cadaveric specimens were used to evaluate the feasibility of the use of a Shark Screw tendon for humeral fixation on both medial and lateral sides. Cyclic loading and failure testing were performed to assess time-zero fixation characteristics and failure load.

**Results:**

A total of sixteen testing scenarios were conducted on the specimens. Implantation was technically feasible without anatomic limitations related to implant size or positioning. Six graft failures occurred (38%), all attributable to poor tissue quality. Tendon slippage was observed in 9 of 16 cases (56%) under progressively increasing axial load, while the Shark Screw tendon remained seated. The mean failure load was 101 N (±50) for medial insertions, 118 N (±77) for lateral insertions, and 110 N (±64) overall. After 100 cycles, tendon displacement averaged 1.1 mm (±0.4) during extension-flexion on the medial side, 1.0 mm (±1.0) on the lateral side, and 1.0 mm (±0.7) overall. During supination-pronation, displacement was 0.8 mm (±0.7) medially, 1.0 mm (±0.8) laterally, and 0.9 mm (±0.7) overall. No anchor pullout was observed, although one anchor tip fractured during insertion.

**Conclusion:**

The Shark Screw tendon appears to be a feasible biologically integrative alternative for humeral fixation in medial and posterolateral elbow ligament re-construction, demonstrating preliminary time-zero fixation characteristics in a cadaveric model. Further biomechanical and clinical studies are needed to confirm long-term outcomes.

Elbow stability is maintained through the combined contribution of osseous congruity, capsuloligamentous structures, and dynamic muscular stabilizers, with the medial and lateral collateral ligament (LCL) complexes serving as key static soft-tissue stabilizers. Posterolateral rotatory instability is the most frequent symptomatic instability pattern and results from insufficiency of the LCL complex, particularly the lateral ulnar collateral ligament (LUCL).^8^^,^^28^ Medial instability, in contrast, reflects insufficiency of the ulnar collateral ligament (UCL) and is commonly caused by repetitive valgus overload, trauma, or iatrogenic injury.^11^ Both patterns can lead to pain, mechanical symptoms, and progressive degenerative changes if untreated.^15^

When conservative treatment fails, surgical ligament re-construction is indicated to restore joint stability in a chronic pattern. Various graft options have been described for LUCL and UCL re-construction, including autologous triceps, palmaris longus, gracilis, or semitendinosus tendons,^9^^,^^10^^,^^30^ with the triceps and palmaris longus being the most frequently used options. Fixation is typically achieved using interference screws, suture anchors, or trans-osseous tunnels.^6^ Despite these established methods, recurrence rates of 8%-12% have been reported after primary re-construction, with graft failure and fixation loosening as the most frequent complications.^3^^,^^16^

Recent clinical data highlight that humeral fixation failure is the predominant cause of recurrent posterolateral instability. A multi-centric analysis by Lappen et al^24^ demonstrated that graft insufficiency or loosening occurred in 59.5% and humeral graft rupture in 37.8% of revision cases following LUCL re-construction, while fixation failure due to drill-hole widening or loosening was observed in nearly half of all patients. These findings confirm that the humeral insertion site represents the critical weak point of LUCL re-constructions. Revision surgery in such cases is technically demanding, often complicated by scarred soft tissues, osteolysis, and bone tunnel overlap, and may require staged bone grafting.^4^ Consequently, functional outcomes after revision re-construction remain only moderate, with complication rates exceeding 20%.^24^

Comparable challenges are seen in revision UCL re-construction, where humeral or ulnar tunnel widening, anchor loosening, and graft attenuation are the principal causes of recurrent valgus instability.^14^^,^^18^ Revision rates of 5%-15% have been reported, frequently accompanied by medial epicondyle osteolysis, heterotopic ossification, or persistent instability.^7^^,^^32^ Biomechanical studies have shown that revision constructs achieve lower initial fixation strength and inferior tendon-to-bone incorporation compared to primary procedures.^21^ These observations emphasize the need for biologically integrative fixation approaches that may be applicable in compromised bone conditions. Previous studies have demonstrated osseous ingrowth and re-modeling of cortical bone screws.^17^^,^^19^^,^^22^^,^^23^^,^^29^^,^^31^ These properties may be of potential interest in revision settings with limited bone stock and warrant further investigation.

The Shark Screw tendon (surgebright GmbH, Lichtenberg, Linz, Austria)—as a representative of a bone tenodesis screw—introduces a novel approach to humeral fixation and is intended to combine mechanical fixation with the potential for biological integration. The implant preserves native Haversian and Volkmann canals, enabling osteoconductive ingrowth and gradual re-modeling into autologous bone through creeping substitution. This results in a broad bone-to-implant interface and secure intra-osseous anchorage without the need for metal hardware.^31^

The present study aimed to evaluate the anatomic feasibility and time-zero fixation characteristics of the Shark Screw tendon for humeral fixation in both medial and posterolateral elbow ligament re-construction procedures. We hypothesized that anatomic re-construction of the medial and posterolateral ligament complexes of the elbow using autografts—with ulnar fixation by Endobutton (Arthrex; Inc., Naples, FL, USA) and humeral fixation using the Shark Screw tendon—would be technically feasible and demonstrate measurable initial fixation characteristics.

## Methods

This is an experimental anatomic and biomechanical feasibility study evaluating humeral graft fixation using the Shark Screw tendon in eight fresh-frozen human cadaveric elbow specimens. Each of the eight cadaveric elbows underwent both lateral and medial re-construction procedures, resulting in 16 re-construction scenarios. To maintain a standardized testing sequence, lateral re-construction and biomechanical testing were consistently performed first, followed by medial re-construction. All specimens were obtained from body donors who willingly donated their bodies to the Medical University of Vienna. Only specimens without evidence of advanced osteoarthritis were used in this study. The study was approved by the ethics committee of the Medical University of Vienna (No. 2384/2025).

### Anatomic assessment and surgical technique

The experimental procedure varied in terms of surgical exposure and ulnar re-fixation technique, providing improved visualization and enabling isolated ulnar anchorage with reduced methodological bias. The principal objective of the cadaveric setup was to assess the feasibility of the alternative humeral fixation approach.

As part of the feasibility study, the skin around the elbow was removed. Looking at the entire triceps tendon, two tendon strips were harvested from the radial and ulnar portions, each measuring 7-10 cm in length and approximately 5 mm in diameter. Both ends of each graft were sutured using the Krakow technique with Ethibond sutures (Johnson & Johnson, New Brunswick, NJ, USA), attaching an Endobutton for ulnar fixation, and a FiberWire 2-0 (Arthrex, Inc., Naples, FL, USA) was used with the Shark Screw tendon for humeral fixation. The prepared tendon grafts were kept in saline-soaked compresses until further use.

### Lateral-side preparation

Using the Kocher approach, the humeral origin of the LUCL was exposed. Next, the native LUCL was detached from its humeral origin to simulate ligament insufficiency before the re-construction was performed and therefore providing full visualization of the entire circumference of the capitellum. Subcutaneous dissection of the anconeus muscle was carried out without further opening of the interval, allowing exposure of the proximal ulna at the junction between the radial head and neck. Primary ulnar fixation was performed through the supinator crest at the native insertion site of the LUCL using an Endobutton. In contrast to the clinical procedure, a 2.7-mm transulnar drilling was performed for extra-luminal placement of the Endobutton together with the reinforcement sutures of the graft, thereby avoiding intra-luminal positioning and ensuring reliable protection against dislocation. The graft was passed proximally, including the reinforced sutures, and the isometric insertion point on the humerus was identified. A 2.0-mm K-wire was temporarily inserted into the distal humerus at the presumed isometric origin of the LUCL, parallel to the joint surface. The graft sutures were positioned around this reference point, and the elbow was cycled through flexion and extension to assess changes in graft tension throughout the range of motion. If near-isometric behavior was observed, the K-wire position was accepted as the definitive humeral fixation site. Then the graft length was determined, shortened to the appropriate length, and reinforced with an additional non-absorbable FiberWire 2-0. Graft thickness was measured for documentation. After confirming the humeral re-fixation point, the K-wire was repositioned *obliquely* in an ascending direction. The K-wire was then overdrilled with a 5.0-mm cannulated drill to a depth of approximately 1.5-2 cm. The superficial cortex was further widened to 5.5 mm to facilitate insertion of the graft and allogeneic bone screw construct. In addition, a 2.7-mm trans-osseous counter-drill hole was created proximally to allow passage of the reinforcement sutures. This enabled secure retrieval of the sutures, controlled graft tensioning, and maintenance of graft preload during final fixation. The construct was inserted into the prepared tunnel, and the sutures were routed proximally, ensuring sufficient length of reinforcement for secure fixation with the human allogeneic bone screw (Fig. 1).

**Figure 1:**
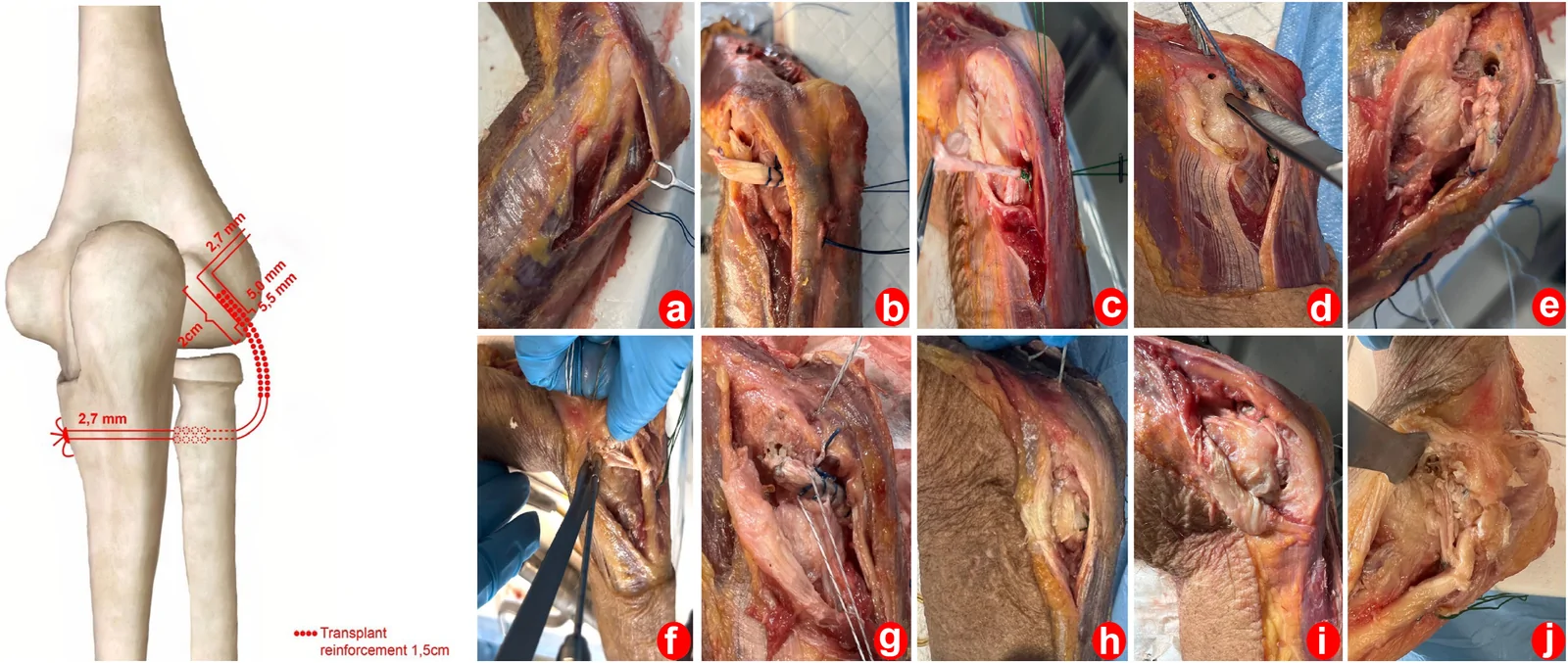
Schematic representation of the experimental procedure for LUCL re-construction with humeral re-fixation using the Shark Screw tendon. (**A**) Exposure of the Kocher interval. (**B**) Identification of the ulnar insertion of the LUCL at the radiocapitellar head-neck junction. (**C**) Ulnar re-fixation of the tendon autograft using an Endobutton. (**D**) Determination of the isometric humeral insertion point, overdrilling with a 5.0-/5.5-mm drill, and creation of a cranial counter-bore for passage of the reinforcement sutures. (**E**) Cranial retrieval of the reinforcement sutures and pulling the graft into position. (**F**) Fixation using the Shark Screw tendon. (**G-J**) Four additional examples of LUCL re-fixation using the Shark Screw tendon. *LUCL*, lateral ulnar collateral ligament.

### Medial-side preparation

Following a standard flexor carpi ulnaris split approach, the ulnar nerve was identified and traced to its entry point between the flexor carpi ulnaris heads. The brachialis muscle was then bluntly dissected from the joint capsule and—using a hook retractor—mobilized together with the flexor-pronator mass. This provided exposure of the ventral capsule, which was subsequently incised to achieve clear visualization of the distal humerus, proximal ulna, the medial collateral ligament (MCL), and the coronoid process. Following exposure, the native medial ulnar collateral ligament (MUCL) was resected to simulate ligament insufficiency before graft re-construction. Distal fixation was performed using an Endobutton together with the reinforcement sutures of the graft, just like on the lateral side, through a transulnar horizontal 2.7-mm drill hole centered on the sublime tubercle. The tendon graft including reinforced sutures was then passed proximally toward the distal humerus. A 2.0-mm eyelet K-wire was inserted at the alleged isometric point *parallel* to the joint surface with perforation of the opposite cortex. Isometric positioning was confirmed by moving the elbow through flexion and extension, ensuring constant graft tension throughout the range of motion. Hereafter, graft length was adjusted, the construct reinforced with an additional non-absorbable FiberWire 2-0, and the resulting tendon diameter measured. The K-wire was then overdrilled with a 5.0-mm cannulated drill to a depth of approximately 1.5-2 cm in the sense of a blind hole without perforation of the joint surface. The superficial cortex was further widened with a 5.5-mm drill to facilitate graft insertion. The graft was then drawn through the humeral tunnel through a loop wire to exit the opposite cortex and secured within the tunnel using the human allogeneic bone screw (Fig. 2).

**Figure 2:**
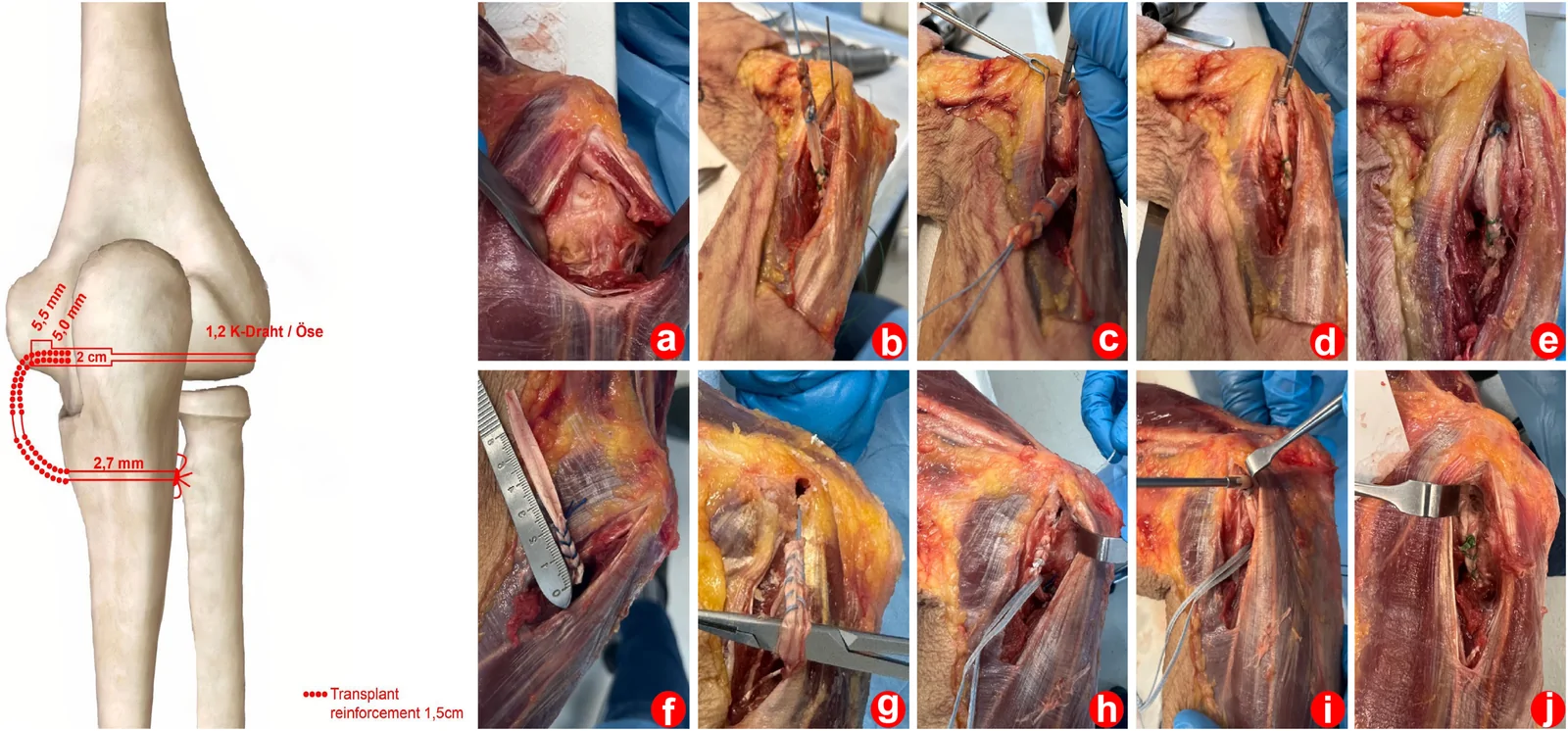
Schematic representation of the experimental procedure for MCL re-construction with humeral re-fixation using the Shark Screw tendon. (**A**) Flexor split following prior exposure of the ulnar nerve. (**B**) Ulnar re-fixation of the graft analogously to the technique used on the radial side. (**C**) After determining the isometric insertion point, creation of a socket (5.0/5.5 mm) using a pre-positioned wire loop. (**D**) Insertion of the Shark Screw tendon while maintaining graft tension. (**E**) Final construct. (**F** and **J**) Four additional examples of MCL re-fixation using the Shark Screw tendon. *MCL*, medial collateral ligament.

For both medial and lateral re-constructions, graft tension was adjusted manually during isometric positioning and fixation. No dedicated graft tensioning or pre-conditioning device was used before biomechanical testing.

The first part of the study presented deals with the technical implementation and feasibility of humeral re-fixation using Shark Screw tendons instead of conventional anchoring techniques (tenodesis screw and trans-osseous drill channels). Taking into account the lack of biological consolidation or bone integration, the second part of the study aimed to assess time-zero fixation characteristics. To explore this question, the study examined possible displacement of the graft in the area of humeral insertion and measured the force required to pull out the graft or generate another failure mechanism. For this purpose, the graft was marked directly at the entrance to the humeral bone drill channel. After that, the study examined whether the position of the transplant or the anchor changed after 100 cycles of extension-flexion and supination-pronation by determining the distance between the mark and the entrance to the drill channel using an electronic measuring device (Vogel, Kevelaer, Germany; 150 × 0.01 mm). To determine failure load, axial tension was applied manually along the longitudinal axis of the graft using a calibrated force gauge, with force recorded in Newtons (N) to a resolution of 0.1 N. Load was increased continuously until construct failure occurred. Failure was defined as graft rupture, tendon slippage, or implant-related complications. The force recorded at the first failure event was documented as the failure load. The values determined were documented in tabular form. For descriptive statistical analysis, mean values and standard deviations were calculated for tendon displacement and failure load. Given the exploratory feasibility design, the small sample size, and the paired nature of the medial and lateral re-constructions performed within the same specimens, no inferential statistical comparisons between medial and lateral fixation procedures were performed.

## Results

Except for one re-construction in which an oversized humeral drill hole prevented secure fixation (specimen 1, medial, Table I), stable anchoring of the allogeneic bone screw was achieved in all specimens.

**Table I:** Specimen characteristics and mechanical test results

Specimen and reconstruction site	Drill-hole displacement (mm) during extension-flexion	Drill-hole displacement (mm) during supination-pronation	Failure load (N)	Failure mode/Technical limitation
Specimen 1 medial	_	_	_	Humeral drill hole oversized
Specimen 2 medial	1.1	1.3	146	Tendon slippage
Specimen 3 medial	1.3	1.7	112	Graft rupture
Specimen 4 medial	1.4	1.3	44	Tendon slippage
Specimen 5 medial	1.1	1.1	107	Anchor-tip fracture, tendon slippage
Specimen 6 medial	0.9	0.0	137	Graft rupture
Specimen 7 medial	1.5	0.4	21	Graft rupture
Specimen 8 medial	0.3	0.0	143	Tendon slippage
Mean and standard deviation	1.1 (±0.4)	0.8 (±0.7)	101 (±50)	
Specimen 1 lateral	2.8	1.8	189	Anchor-tip fracture, tendon slippage
Specimen 2 lateral	1.5	1.3	31	Graft rupture
Specimen 3 lateral	1.2	1.8	29	Tendon slippage
Specimen 4 lateral	1.4	1.6	224	Tendon slippage
Specimen 5 lateral	1.1	1.1	118	Tendon slippage
Specimen 6 lateral	0.0	0.0	41	Graft rupture
Specimen 7 lateral	0.0	0.0	127	Tendon slippage
Specimen 8 lateral	0.0	0.0	181	Graft rupture
Mean and standard deviation	1.0 (±1.0)	1.0 (±0.8)	118 (±77)	

Medial = parallel drill hole. Lateral = oblique drill hole.

Failure mode analysis demonstrated that graft rupture occurred in 6 of 16 re-constructions (38%), which was attributed to poor tendon quality of the cadaveric grafts. Tendon slippage occurred in 9 of 16 re-constructions (56%) under increasing axial load. Importantly, no loosening, displacement, or pullout of the allogeneic bone screw was observed in any specimen.

The mean failure load was 101 N (±50) for medial insertions, 118 N (±77) for lateral insertions, and 110 N (±64) overall.

Following 100 cycles of extension-flexion, the mean tendon displacement from the humeral fixation site was 1.1 mm (±0.4) on the medial side, 1.0 mm (±1.0) on the lateral side, and 1.0 mm (±0.7) overall. After 100 cycles of supination-pronation, average displacement measured 0.8 mm (±0.7) medially, 1.0 mm (±0.8) laterally, and 0.9 mm (±0.7) overall.

In one specimen (6%), the tip of the anchor fractured during insertion due to a lever mechanism; the anchor was subsequently seated using gentle impaction with a surgical mallet.

## Discussion

The present study investigated the anatomic and biomechanical feasibility of using an allogeneic cortical bone screw as an alternative method of humeral fixation for both LUCL and MCL re-construction in cases of posterolateral and medial elbow instability. The Shark Screw tendon, a biologically integrative implant composed of human cortical bone, represents a novel alternative to conventional metal or bioabsorbable fixation devices. Clinical series in small joints have shown that allogeneic cortical bone screws can fully re-model into host bone via creeping substitution, obviating hardware removal and showing low implant-related complication rates.^22^ The results of this study suggest that fixation using the Shark Screw tendon is technically feasible and demonstrates time-zero fixation characteristics under controlled laboratory conditions. Early clinical cohorts beyond the hand also report favorable radiologic and clinical integration of allograft screws, supporting the biological fixation concept that underlies our approach.^17^ However, the present study was not intended to evaluate long-term biological integration. Because the cadaveric model does not permit tendon-to-bone healing, osseous re-modeling, or creeping substitution of the allogeneic bone screw, conclusions regarding long-term implant incorporation and clinical durability cannot be drawn from the present data. Future in vivo studies and clinical investigations are required to determine whether these biological properties translate into improved long-term outcomes.

As an exploratory feasibility study, no conclusions regarding superiority, equivalence, or inferiority to established fixation systems can be drawn.

Anatomic feasibility was confirmed in all but one specimen, where failure resulted from overdrilling of the humeral cortex, underscoring the importance of a precise surgical technique. No anatomic constraints were observed regarding the implant's dimensions (5 mm diameter and 15 mm length) at the isometric origins of the LUCL and MCL. Adequate exposure and graft placement were consistently achieved through standard surgical approaches, without relevant anatomic constraints.

An essential aspect of successful collateral ligament re-construction of the elbow is the identification and restoration of isometric attachment points on the humerus. These points represent anatomic regions where the distance between the humeral and ulnar ligament insertions remains nearly constant throughout the elbow's range of motion. Achieving isometry minimizes graft length changes during flexion and extension, thereby reducing tension peaks, graft elongation, or failure.^12^^,^^27^ For the MUCL, particularly its anterior bundle, the optimal humeral isometric point lies at the anteroinferior aspect of the medial epicondyle, slightly anterior to its center and close to the native UCL origin.^2^^,^^26^ For the LUCL, the humeral isometric point is located slightly anterior and distal to the center of the lateral epicondyle, near the origin of the LCL complex.^25^^,^^27^

In revision cases, identifying these native isometric landmarks is often challenging because of bone loss, tunnel widening, or previous hardware removal, making biologically integrative fixation systems potentially relevant in this setting. Whether the Shark Screw tendon provides clinical advantages in this setting remains to be investigated.

Biomechanical assessment demonstrated mean tendon displacements of 1.1 mm (±0.4) medially, 1.0 mm (±1.0) laterally, and 1.0 mm (±0.7) overall after 100 cycles of extension-flexion. Following 100 cycles of supination-pronation, displacements of 0.8 mm (±0.7) medially, 1.0 mm (±0.8) laterally, and 0.9 mm (±0.7) overall were observed. The observed displacement remained small throughout cyclic testing. Cyclic displacement and related measures of construct laxity have been used in previous biomechanical studies to characterize the mechanical behavior of soft-tissue and ligament re-construction constructs.^1^^,^^13^^,^^20^ The mean failure load was 101 ± 50 N for medial fixation, 118 ± 77 N for lateral fixation, and 110 ± 64 N overall. For contextual comparison, a previous biomechanical anterior cruciate ligament study reported similar graft slippage under cyclic loading between an allogeneic cortical bone screw and a conventional interference screw.^5^ However, these findings were obtained in a different anatomic and experimental setting and cannot be directly extrapolated to elbow ligament re-construction. Furthermore, the relatively high standard deviation and isolated low-load failures emphasize the influence of graft and implant quality on the observed behavior of the construct.

The observed failure modes are important for interpretation of the present findings. Although no implant loosening or pullout occurred, tendon slippage was observed in more than half of the re-constructions. These findings suggest that the behavior of the construct depended more on graft quality and tendon-suture interaction than on implant fixation. These findings support the technical feasibility of the concept but also highlight the need for further optimization and comparative biomechanical testing.

Proximal reinforcement of the graft with an intra-osseous portion of approximately 1.5 cm appears beneficial in this context. Six failures (38%) were attributed to graft rupture, most likely due to compromised tendon quality, reflecting the variability inherent to cadaveric tissue. In one instance, anchor-tip fracture occurred during insertion, highlighting the importance of careful handling, avoidance of excessive torque, and manual rather than powered insertion. Pre-drilling the superficial cortex to 5.5 mm was found to facilitate smooth insertion and reduce stress on the implant.

The biological properties reported for cortical bone screws in previous clinical studies, including biological re-modeling into host bone and the potential to avoid secondary hardware removal, suggest that this concept may warrant further investigation for ligament re-construction.^23^ These properties may also be of particular interest in revision settings, where compromised bone stock can present an additional challenge. However, the present cadaveric feasibility study was not designed to evaluate these potential clinical advantages. This re-modeling capability and the absence of implant-related complications have been documented in distal interphalangeal arthrodesis cohorts using cortical bone screws, with complete integration on follow-up.^22^ Beyond small joints, non-union series in the upper limb report high union rates and suggest fewer hardware-related issues when allograft screws are used as part of stability-plus-biology strategies—evidence that aligns with our rationale for revision elbows with compromised bone stock.^19^ Similarly, scaphoid fracture/pseudarthrosis cohorts have shown union rates comparable or superior to conventional techniques, with the added benefit of avoiding secondary hardware removal, again reinforcing the “fix bone with bone” paradigm.^29^

To the best of our knowledge, this is the first study to evaluate the use of a human allogeneic bone screw in ligament re-construction of the elbow, introducing a biologically integrative concept for humeral fixation in both primary and revision settings, the potential clinical benefits of which remain to be demonstrated.

## Limitations

This study has several limitations. First, the small sample size and the use of cadaveric tendon tissue with variable biological quality limit the generalizability of these findings. As this study was conceived as an exploratory feasibility investigation, no formal a priori power analysis was performed. The study was designed to assess surgical applicability and initial fixation behavior rather than to perform confirmatory comparisons between medial and lateral fixation constructs. Given the small sample size and the paired nature of the medial and lateral re-constructions performed within the same specimens, inferential statistical comparisons between medial and lateral fixation procedures were not performed, and the results are presented descriptively. Several failures were attributable to graft rupture, suggesting that the construct’s performance may have been influenced by tissue quality rather than fixation characteristics alone. Although native tendon grafts were selected to reproduce the clinical re-construction scenario, future studies using standardized synthetic grafts or uniform allograft tissue may allow a more isolated assessment of implant fixation behavior.

Second, the absence of in vivo biological processes, such as bone re-modeling, tendon-to-bone healing, and creeping substitution of the allogeneic bone screw, precludes direct conclusions regarding long-term stability, biological integration, or clinical durability. Therefore, the present study should be interpreted as a time-zero feasibility and initial fixation study rather than an evaluation of long-term implant performance.

Third, cyclic loading was limited to 100 cycles and does not reflect physiological repetitive stress over time. Furthermore, both medial and lateral re-constructions were performed on each cadaveric specimen. Although the primary outcome measures focused on the local fixation behavior at the humeral insertion site, this approach may have altered the native biomechanical environment of the elbow and does not fully replicate the clinical scenario of isolated LUCL or MUCL re-construction. In addition, no standardized graft pre-conditioning or tensioning protocol was applied before fixation. Consequently, variability in tendon creep and intrinsic graft properties may have contributed to the observed displacement and failure characteristics. Future biomechanical studies should consider standardized graft tensioning to improve reproducibility.

Fourth, the lack of a comparative control group using established fixation devices such as titanium, bio-composite, or polyether ether ketone interference screws restricts the ability to directly quantify the biomechanical performance of the allogeneic bone screw. Because the present study was designed as a feasibility investigation rather than a comparative biomechanical superiority study, no conclusions can be drawn regarding superiority, equivalence, or inferiority relative to currently available fixation systems. These findings should be interpreted as proof-of-concept feasibility data rather than evidence of biomechanical superiority over established fixation techniques. Future comparative biomechanical investigations are warranted.

Finally, the Shark Screw tendons used in this study were provided by the manufacturer, surgebright GmbH, which also granted access to laboratory facilities. Although the manufacturer had no involvement in the study design, data collection, data analysis, interpretation of the results, manuscript preparation, or decision to submit the manuscript for publication, this industry support represents a potential source of bias and should be considered when interpreting the findings.

Despite these limitations, the study consistently demonstrated technical feasibility of implantation and absence of implant pullout or loosening, supporting continued biomechanical evaluation and subsequent clinical investigation.

## Conclusion

The results of this feasibility study demonstrate anatomic feasibility and preliminary time-zero fixation characteristics of the Shark Screw tendon for humeral fixation in medial and posterolateral elbow ligament re-construction. Implantation was technically feasible in all specimens, and biomechanical testing demonstrated limited micro-motion and the absence of implant pullout under the tested conditions. Because this study was designed as a time-zero feasibility investigation without a comparative control group, no conclusions can be drawn regarding superiority or long-term clinical performance. Further comparative biomechanical and clinical studies are warranted.

## Disclaimers:

Funding: This study received in-kind support from surgebright GmbH (Austria), which provided the Shark Screw tendons used in the experimental setup and granted access to laboratory facilities. No direct financial funding or additional external grants were received. The company had no involvement in the study design, data collection, data analysis, interpretation of the results, manuscript preparation, or decision to submit the manuscript for publication. Cadaveric specimens were provided by the Medical University of Vienna (Lukas F. Reissig, MD, PhD).

Conflicts of interest: The authors, their immediate families, and any research foundations with which they are affiliated have not received any financial payments or other benefits from any commercial entity related to the subject of this article.

## Data availability statement

The data that support the findings of this study are openly available in Figshare (reference number: 10.6084/m9.figshare.31436875).

## Ethics declaration

Written informed consent to take part in the study and to publish the article has been obtained from all participants or their legal representatives. The privacy rights of participants have been observed.

This study includes human biological material; hence, consent was obtained from donors, or their next of kin or legal representatives, for use in this study and for publication of the article. The samples used in this research were not sourced from executed prisoners or prisoners of conscience.

This study was performed in compliance with relevant laws, regulatory frameworks and guidelines where the research took place.
